# FlashLightNet: An End-to-End Deep Learning Framework for Real-Time Detection and Classification of Static and Flashing Traffic Light States

**DOI:** 10.3390/s25206423

**Published:** 2025-10-17

**Authors:** Laith Bani Khaled, Mahfuzur Rahman, Iffat Ara Ebu, John E. Ball

**Affiliations:** Department of Electrical and Computer Engineering, James Worth Bagley College of Engineering, Mississippi State University, Starkville, MS 39762, USA; mr2527@msstate.edu (M.R.); ie93@msstate.edu (I.A.E.)

**Keywords:** neural network, YOLO, traffic light detection, LSTM, ResNet, machine learning, flashing traffic light recognition

## Abstract

Accurate traffic light detection and classification are fundamental for autonomous vehicle (AV) navigation and real-time traffic management in complex urban environments. Existing systems often fall short of reliably identifying and classifying traffic light states in real-time, including their flashing modes. This study introduces FlashLightNet, a novel end-to-end deep learning framework that integrates the nano version of You Only Look Once, version 10m (YOLOv10n) for traffic light detection, Residual Neural Networks 18 (ResNet-18) for feature extraction, and a Long Short-Term Memory (LSTM) network for temporal state classification. The proposed framework is designed to robustly detect and classify traffic light states, including conventional signals (red, green, and yellow) and flashing signals (flash red and flash yellow), under diverse and challenging conditions such as varying lighting, occlusions, and environmental noise. The framework has been trained and evaluated on a comprehensive custom dataset of traffic light scenarios organized into temporal sequences to capture spatiotemporal dynamics. The dataset has been prepared by taking videos of traffic lights at different intersections of Starkville, Mississippi, and Mississippi State University, consisting of red, green, yellow, flash red, and flash yellow. In addition, simulation-based video datasets with different flashing rates—2, 3, and 4 s—for traffic light states at several intersections were created using RoadRunner, further enhancing the diversity and robustness of the dataset. The YOLOv10n model achieved a mean average precision (mAP) of 99.2% in traffic light detection, while the ResNet-18 and LSTM combination classified traffic light states (red, green, yellow, flash red, and flash yellow) with an F1-score of 96%.

## 1. Introduction

The evolution of AV technologies has significantly transformed the landscape of transportation, promising safer roads and more efficient travel. However, to realize this vision, vehicles must reliably interpret road signs [1,2,3] and traffic lights [4,5,6] in real time, an essential yet challenging task in dynamic and unpredictable urban environments. Although significant research has focused on traffic light recognition, largely addressing static signals (red, green, and yellow), the classification of flashing signals, which frequently indicate caution or emergency scenarios, remains understudied [6,7,8,9,10]. Unlike static signals that maintain consistent illumination, flashing lights pose unique challenges due to their intermittent visual presentation, which can be further complicated by variable lighting conditions, occlusion, camera angles, and background clutter [11,12]. Traditional frame-by-frame classification methods are ineffective for this task, as they lack the temporal awareness needed to distinguish between a flashing signal and other brief visual disturbances. Moreover, most existing datasets and models overlook flashing signals entirely, focusing instead on static-state classification. This gap highlights the need for frameworks that can model temporal aspects and support real-time decision-making, especially in high-stakes situations where flashing signals are common.

Despite the importance of signalized intersections for traffic safety, approximately one-third of all intersection fatalities in the United States occur at these locations, which account for approximately 14% to 25% of all fatal crashes nationally [13]. However, few studies have explicitly examined flashing signal indications, such as flashing arrows, flashing red, or flashing yellow, as a central focus of their analyses. The limited research available shows promising results in certain contexts. For instance, evaluations of flashing signal indications at unsignalized intersections have demonstrated their effectiveness; data from North Carolina and South Carolina showed a 13.3% reduction in angle crashes and a 10.2% reduction in fatal crashes. Even conservative estimates from this study indicated notable reductions of at least 4% for angle crashes and 1% for injury and fatal crashes [14]. Meanwhile, survey-based studies focusing on the flashing yellow arrow (FYA) have revealed substantial comprehension issues. In one study, only about 52% of respondents correctly interpreted the expected behavior for a related signal (the red arrow) in permissive turn settings, suggesting that nearly half of drivers may misunderstand specific flashing signal indications [15]. This research gap is particularly significant. Although flashing signals have been shown to influence safety outcomes in certain scenarios, such as unsignalized intersections, their broader role and drivers’ comprehension at signalized intersections remain largely unexplored in the literature.

While the long-term vision for autonomous transportation rightly emphasizes direct vehicle-to-infrastructure (V2I) communication—where traffic lights transmit their status digitally—the vast majority of current traffic lights are traditional and non-communicative [16,17,18]. The transition to a fully connected infrastructure will be costly and time-consuming, likely lasting decades [19,20,21]. During this long transition period, AVs must retain strong perceptual capabilities to visually interpret traffic lights and ensure safe operation [22,23]. Our work directly addresses this transitional challenge. Furthermore, even in a V2I-enabled future, perception remains indispensable as an additional safeguard against communication failures, cyberattacks, or data inaccuracies [20,24]. Therefore, developing visual traffic light recognition—especially for complex time-based situations such as flashing signals—is a fundamental step toward achieving reliable and safe autonomous navigation.

To bridge this critical gap, we have proposed FlashLightNet, a comprehensive traffic light detection and classification framework developed to recognize both static and flashing signals effectively. This approach utilizes state-of-the-art deep learning technologies, integrating three complementary modules: YOLOv10 [25] for precise real-time object detection, ResNet-18 [26] for robust extraction of spatial features, and LSTM [27] networks for modeling temporal patterns essential in classifying flashing states.

In support of this framework, a custom dataset has been developed specifically for this study, comprising video recordings from a diverse range of intersections in Starkville, Mississippi, and the Mississippi State University campus, along with simulation-based video datasets with different flashing rates—2, 3, and 4 s—of traffic light states generated using RoadRunner, a simulation tool. The dataset includes five distinct traffic light classes: red, green, yellow, flash red, and flash yellow, and has been carefully annotated to preserve frame sequences, enabling robust temporal training. By capturing a wide range of real-world conditions, including variable lighting and environmental factors, the dataset provides a valuable foundation for training and developing models with generalization capabilities in practical applications.

Through rigorous experimental validation, our proposed model has demonstrated an outstanding performance, achieving a mAP of 99.2% in detecting traffic lights and high accuracy in classifying both static and flashing states. Comparative analyses further highlight that our integrated spatial–temporal approach significantly outperforms existing methods, effectively addressing scenarios previously unexplored or inadequately handled by current models. The results of this study show a significant advancement in AV systems, providing a strong foundation for safer intersection navigation and enhanced driver assistance technologies. By effectively interpreting complex traffic signals under diverse real-world conditions, our framework brings autonomous driving systems a step closer to achieving the reliability required for safer intersection navigation. We can summarize the contributions of our work as follows:

1. Unified Detection and Classification Framework: We propose FlashLightNet, a unified end-to-end deep learning pipeline to detect and classify traffic lights, including both static and flashing states, by incorporating YOLOv10n for detection, ResNet-18 for spatial feature extraction, and LSTM for temporal classification of traffic light states. To the best of our knowledge, this is the first study to comprehensively address flashing traffic light recognition within a unified system.
2. Comprehensive Benchmarking and Comparative Evaluation: Extensive experiments were conducted to develop the proposed system, including comparisons of multiple YOLOv10 versions, various feature extractors (ResNet-18, MobileNetV3, EfficientNet-B0), and different temporal models (LSTM, Gated Recurrent Unit (GRU), vanilla Recurrent Neural Network (RNN)). Additional evaluations considered the number of LSTM layers, optimizer types, and learning rates. The final configuration was validated and demonstrated the best trade-off between accuracy and computational efficiency, making it suitable for real-time deployment.
3. Better Performance and Real-Time Capability: The proposed framework achieves state-of-the-art results in both real and simulated environments, with an mAP of 99.2% for traffic light detection and an F1-score of 96% for classification. Designed with computational efficiency in mind, the system supports real-time performance, defined here as processing video at 50 frames per second (fps) using a GPU setup consisting of NVIDIA A100, ensuring seamless integration with autonomous driving systems where latency-critical decisions are required. This demonstrates both the robustness and practicality of the framework for deployment in autonomous driving applications.

The remainder of this paper is organized as follows: Section 2 reviews the related work on traffic light detection and classification and different models or methods used for that purpose; Section 3 details our research methodology, including dataset construction, model design, and training strategy; Section 4 describes the experimental setup; Section 5 presents and discusses the experimental results; and Section 6 concludes the study with future research directions.

## 2. Related Work

In this section, we review the relevant literature in the field of traffic light detection and classification, especially focusing on recent deep learning-based frameworks. We first discuss general traffic light detection approaches and their limitations, followed by an analysis of deep learning models used for spatial feature extraction and classification. Subsequently, we introduce various temporal modeling alternatives, including LSTM, Temporal Convolutional Networks (TCN), Bayesian and Markov models, fuzzy systems, and reinforcement learning. We conclude by justifying our selection of an LSTM-based architecture for traffic light state classification in dynamic environments.

### 2.1. General Approaches for Traffic Light Detection

General traffic light (TL) detection methods can be broadly categorized into model-based and learning-based approaches [5]. Model-based methods typically rely on heuristically designed rules that incorporate shape, color, and intensity information. One common technique is color density thresholding for red, yellow, and green traffic lights, which has been extensively used across numerous studies [4,11,28,29,30,31,32,33,34]. Additional model-based strategies employ fuzzy clustering [35,36] to represent color features more flexibly and spotlight detection using white top hat operation on grayscale or HSV channels to handle robustness on disturbances in color [37,38]. Shape-based models such as the circular and modified Hough transform [39], Laplacian edge detection [31], and fast radial symmetry [40] are applied to localize circular traffic light components. Binary Large Object (BLOB) analysis is also widely adopted to extract candidate regions based on size, shape, and structural cues, filtering out non-traffic-light elements using features like aspect ratio, contour regularity, and estimated height [41]. Learning-based methods aim to improve robustness by extracting discriminative features and training classifiers. Early approaches used cascading Haar classifiers [42], while more recent works extract features such as color histograms, Histogram of Oriented Gradients (HoG) [43], Local Binary Patterns (LBP) [31], Gabor filters [44], and geometric descriptors [45]. These features are then classified using techniques such as Support Vector Machines (SVM) [32], JointBoost [45], or neural networks [42]. Some systems combine multiple cues such as color, shape, structure, and contextual location information to increase the classification accuracy [38].

While model-based and learning-based traffic light detection techniques have demonstrated strong performance under controlled conditions, they face several challenges in real-world environments. Color-based models are highly sensitive to illumination changes, shadows, and sensor variations, often leading to detection failures. Many methods depend on precise blob segmentation, yet the appearance of actual traffic lights can vary significantly across frames, reducing the consistency. Heuristic approaches, including fixed thresholding or template matching, tend to overfit specific scenarios and lack adaptability to new conditions. Learning-based detectors, although more robust, demand large and diverse training datasets to generalize well. Furthermore, systems that rely on GPS-based maps can suffer from false negatives if traffic lights are not pre-mapped, such as during road construction or dynamic route changes.

### 2.2. Deep Learning-Based Traffic Light Detection and Classification

Recent studies have demonstrated substantial improvements in detection accuracy using deep learning models such as YOLO (You Only Look Once), Faster R-CNN (Region-based Convolutional Neural Network), SSD (Single Shot Detector), and their variants [46,47,48]. For instance, Ennahhal et al. [49] used YOLOv3 on the Bosch and LISA datasets to enhance recognition speed and precision. Some approaches also fuse shallow and deep features for better small-object detection, as seen in YOLOv4 extensions using Gaussian bounding box uncertainty models [50]. Advanced frameworks have integrated multi-camera setups and GPS data to refine region-of-interest detection. Possatti et al. [51] used prior maps and 3D coordinate projections to recognize relevant traffic lights for autonomous driving routes. Similarly, two-stage detection strategies have been proposed to handle individual bulb localization followed by classification [7,8]. Despite progress, most of these works are limited to static traffic light states and do not address flashing signal patterns or multiple signal types (arrow, countdown).

Although recent deep learning models offer real-time and high-accuracy detection, they face several limitations:

- Most studies exclude flashing signal states, which are crucial for real-world autonomous driving decisions. Many models treat each frame independently, lacking the temporal context necessary for recognizing flashing lights or ambiguous transitions.
- Many frameworks are confined to binary or three-class classification, failing to generalize to diverse signal types such as arrows or countdown timers.

These gaps motivate the integration of temporal modeling techniques into traffic light recognition systems.

### 2.3. Temporal Modeling Techniques in Traffic Applications

Temporal modeling is essential when the task involves sequential state transitions such as detecting flashing traffic lights. Several modeling strategies have been explored in time-series domains:

LSTM networks have shown strong performance in various traffic-related tasks such as vehicle speed prediction, car-following modeling, and driver behavior analysis [52,53,54,55]. Their memory gates allow learning long-term dependencies, which suits tasks involving temporal state changes, such as distinguishing between blinking and static signals. Temporal Convolutional Networks (TCN) offer an alternative to RNNs by employing dilated convolutions across temporal sequences. They are computationally efficient and often train faster than LSTMs, while achieving comparable accuracy in time-series tasks [53,56]. However, their performance on sparse event-driven sequences like flashing lights is not yet fully explored in this context. Dynamic Bayesian Networks (DBNs), Markov Temporal Bayesian Networks (MTBNs), and Temporal Node Bayesian Networks (TNBNs) model time as probabilistic transitions, often assume fixed time intervals, and can become computationally expensive in dynamic scenes [57]. Fuzzy logic-based temporal reasoning, including neuro-fuzzy systems and fuzzy cognitive maps (FCM), handles uncertainty in sequential decision-making. Deep attention-based FCMs have been explored for spatiotemporal modeling in sensor networks and transportation systems [58].

Among the reviewed methods, LSTM stands out as a robust and well-established temporal model. Its architecture is explicitly designed to mitigate vanishing gradients and preserve long-term dependencies through gated memory cells. Prior works in traffic prediction and behavior analysis have validated LSTM’s effectiveness in sequential tasks, where it consistently outperforms classical models like Autoregressive integrated moving average (ARIMA) and even recent temporal networks under noisy or sparse event patterns [52,53,54,55,59]. While TCN offers speed benefits, LSTM’s flexibility in handling irregular or flashing transitions makes it particularly suited for our use case. Furthermore, the hybrid architecture combining ResNet for spatial feature extraction and LSTM for temporal modeling (e.g., ResNet-LSTM) has shown excellent generalizability across domains such as healthcare, structural monitoring, and environmental sensing [60,61,62]. These results reinforce our decision to adopt a YOLOv10n–ResNet18–LSTM framework for traffic light state recognition, particularly when both spatial and temporal features are critical.

Several deep learning–based methods have been proposed for traffic light detection and recognition. Wang et al. [50] reported an mAP of 82.15% for their YOLOv4 traffic lights detection and recognition method. Possatti et al. [51] achieved an mAP of 55.21% with a recall of 62.28% for traffic light recognition using deep learning and prior maps. The VGG16 model with Stochastic Gradient Descent (SGD) optimization by Lin et al. [7] exceeded 90% classification accuracy for traffic light detection and recognition using a two-stage framework. A coarse-to-fine deep learning based framework for traffic light recognition further improved performance, reaching 99.04% precision and 97.04% recall, with a recognition speed of 9.9 ms per frame [8].

Building upon these advances, our research addresses key limitations of existing approaches by proposing an end-to-end framework (FlashLightNet) that integrates YOLOv10n for object detection, ResNet-18 for spatial feature extraction, and LSTM for temporal classification. Unlike previous studies, our model is trained on a comprehensive dataset that includes both static and flashing signals from real-world intersections in Mississippi, as well as simulation-based data. This design enables the accurate real-time classification of diverse traffic light states. FlashLightNet achieves 96% precision, 97% recall, and a 96% F1-score, demonstrating superior performance compared to prior methods and showing strong potential for deployment in intelligent traffic systems, particularly within autonomous driving applications.

Table 1 provides a comparative summary of our proposed model and recent state-of-the-art studies on traffic light detection and recognition using deep learning models. Specifically, the table shows each model’s ability to detect and classify static and flashing traffic lights and whether it operates in real time. The table also lists the dataset used and the corresponding model architecture for each study. Most of the reviewed studies—such as those by Rao et al. [9], Rahman et al. [10], and De Guia et al. [6]—primarily focused on detecting and classifying static traffic light states (i.e., red, green, and yellow) using popular object detection models such as ResNet50, YOLOv7, YOLOv5, and YOLOv8. These studies typically employed public or custom datasets that only included static traffic light states. None of the aforementioned models were capable of recognizing flashing traffic signal patterns—such as flashing red or yellow—which are crucial for real-world autonomous driving systems. Moreover, although several studies, such as those by Niu et al. [63] and Chen and Lin [64], demonstrated real-time detection capabilities, they did not extend their models to handle flashing signal states.

### 2.4. You Only Look Once (YOLO) Version 10

**Figure 1:**
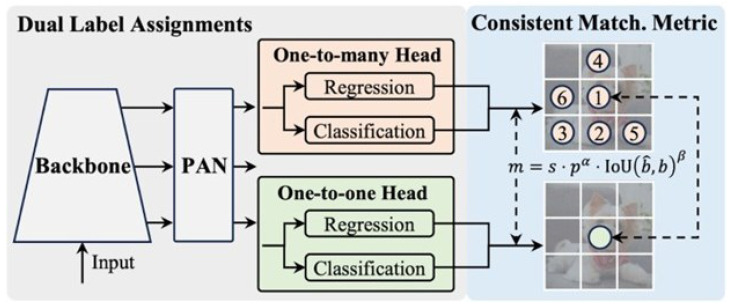
YOLOv10 architecture. Here, *p* is the classification score, $\hat{b}$ and *b* denote the bounding box of the prediction and the instance, respectively, *s* represents the spatial prior, indicating whether the anchor point of the prediction is within the instance, α and β are two hyperparameters that balance the impact of the semantic prediction task and the location regression task, and *m* represents the consistent match metrics. PAN refers to the Path Aggregation Network, which aggregates and propagates features across different levels of the network as a component within the “neck” of the model.

YOLO is one of the latest advanced object detection technologies that relies on a single-stage process for object detection, unlike traditional detection technologies that rely on two stages. It comes in various versions, including nano, small, medium, balanced, large, and extra-large [25]. In this paper, we used a nano version (YOLOv10n) because it strikes a balance between speed and accuracy in real-time applications. The YOLOv10 architecture is composed of four main components: the Backbone, Neck, One-to-Many Head, and One-to-One Head, as illustrated in Figure 1 [25].

### 2.5. Residual Neural Networks 18 (ResNet-18)

**Figure 2:**
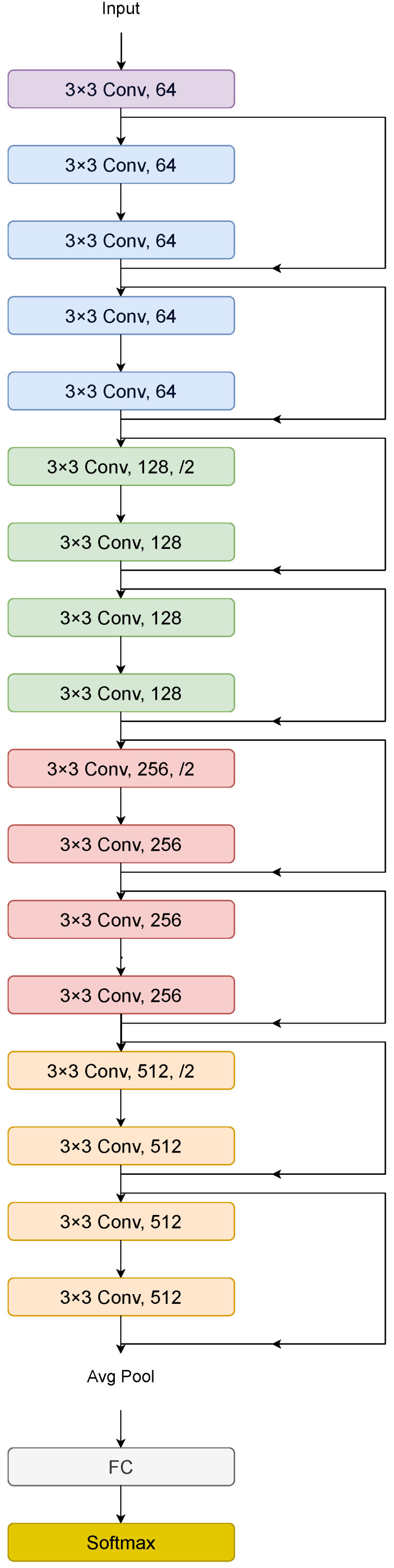
ResNet-18 architecture.

Residual Neural Networks (ResNet), introduced by Kaiming He et al. in 2015 [26], addressed the vanishing gradient problem (VGP) using residual learning, enabling the training of very deep neural networks. This improves the model’s ability to learn complex data patterns. In this paper, we have used ResNet-18 as a feature extractor due to its lightweight architecture, which provides an optimal trade-off between speed and accuracy for real-time applications [26,67]. ResNet-18 consists of 18 layers structured into five stages with residual connections that mitigate gradient degradation, as illustrated in Figure 2 [26,68,69].

### 2.6. Long Short-Term Memory (LSTM)

Long short-term memory (LSTM) is a component of the recurrent neural network (RNN), developed by Hochreiter and Schmidhuber in 1997 to overcome the vanishing gradient problem (VGP) [27]. LSTM is characterized by its ability to capture long-term dependencies between sequential datasets, which makes it ideal for time series applications [27]. The basic architecture of LSTM is referred to as a memory block. Each memory block consists of two cell states and three gates: an input gate that determines what information should be stored or updated in the memory cell, an output gate that controls the output of the memory cell [70], and a forget gate that determines what information should be inserted or removed from the memory block [70].

## 3. Research Methodology

**Figure 3:**
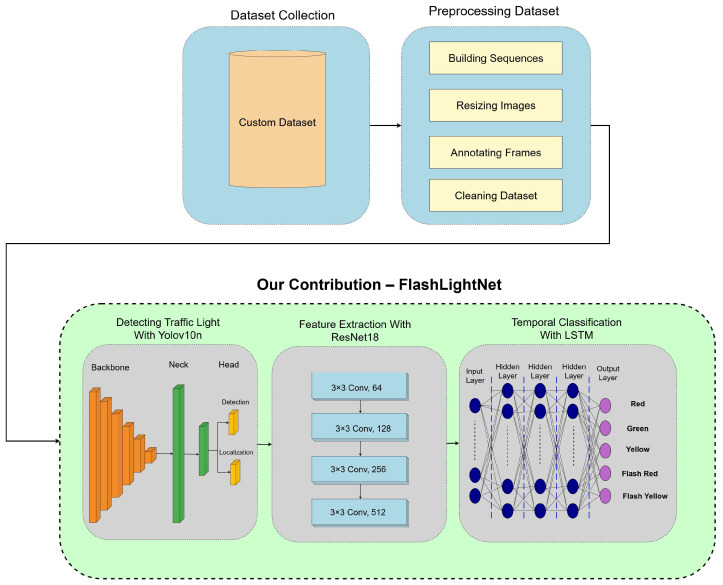
Methodological workflow of the proposed system.

This research presents FlashLightNet, a comprehensive end-to-end framework for traffic light detection and recognition, capable of handling both static and flashing states. The methodology consists of three main components: YOLOv10n for detection, ResNet-18 for feature extraction, and LSTM for temporal state classification. Furthermore, a custom dataset is developed to ensure the robust performance of the proposed approach under various real-world scenarios. Figure 3 illustrates the top-tier methodological workflow of the proposed system.

### 3.1. Data Collection

A comprehensive custom dataset was developed specifically for this study to train and evaluate the proposed FlashLightNet framework. The dataset consists of two complementary parts: real-world recordings and simulation-based videos. The real-world dataset comprises 55 video sequences captured at 50 fps using a Sony a6500 camera (Sony Group Corporation, Tokyo, Japan) at various intersections in Starkville, Mississippi, and the Mississippi State University campus. An additional simulated dataset was generated using RoadRunner to enhance the model’s ability to recognize flashing signals. This controlled simulated environment has enabled the creation of a unique set of videos with precisely defined flashing rates of 2, 3, and 4 s, providing a noise-free foundation for learning fundamental temporal patterns. The combination of real-world data and controlled simulated data creates a robust and diverse benchmark, greatly improving the model’s generalization capabilities in recognizing both static and flashing traffic lights.

#### 3.1.1. Recording Videos for Dataset Creation

The first step involves recording 55 videos at 50 fps using a high-resolution Sony a6500 camera. This frame rate was intentionally selected to capture smooth motion and precise temporal details of traffic light changes, ensuring that no transitions are missed—an essential requirement for accurate blinking detection and classification. The videos were taken at various intersections and lighting conditions, including daytime and evening scenarios, in Starkville, Mississippi, and the campus of Mississippi State University, ensuring a diverse range of intersections. These locations were carefully selected to simulate the actual challenges that AVs might face in real-world environments. In addition, we built a simulation-based video dataset for traffic light states at several intersections using RoadRunner, further enhancing the diversity and robustness of the dataset. This resulting custom dataset provides a strong foundation and reliable platform for developing a reliable and accurate detection and classification model by replicating actual intersection scenarios, including those with flashing red and flashing yellow lights.

#### 3.1.2. Video Segmentation, Frame Categorization, and Dataset Cleaning

The recorded videos, along with the simulation-based videos generated using RoadRunner, were then converted into individual frames. These frames were categorized into five classes: red, green, yellow, flash red, and flash yellow. Particular attention was given to maintaining the sequential order of the frames throughout the cleaning process, as this is important in supporting time-dependent analysis and training, especially in flashing traffic light recognition. Any video or frame that was distorted, blurry, unclear, or did not meet quality standards was removed from the dataset. This step is important to improve the accuracy of the deep learning model during training by removing noise and ensuring the overall integrity and reliability of the data. This methodology ensured clarity and consistency, leading to a well-organized and reliable dataset for training purposes.

#### 3.1.3. Annotating Frames Using Roboflow

After preprocessing and developing the dataset, it was passed through the annotation and labeling process. Roboflow was used to automatically annotate the frames in the YOLOv10 format, in addition to providing normalized bounding box coordinates with parameters like x_center, y_center, width, and height. Consistency and maintaining correct order across the frames were ensured through the automatic annotation process, especially for the flashing lights that were categorized into distinct categories such as “Flash Red” and “Flash Yellow”. The “on” and “off” frames for the flashing lights were classified within their respective flashing categories. Additionally, automated validation scripts were employed to verify the correctness of the annotation process by checking the matching between the traffic light and its corresponding class, ensuring the accuracy of the bounding box coordinates, and detecting any corruption or missing data in the labeled dataset. Any anomalies discovered during this process were manually reviewed and adjusted. This comprehensive quality control strategy ensured the quality and reliability of the dataset, making it a solid foundation for training and evaluating the proposed deep learning model.

#### 3.1.4. Resizing Images for Standardization

To the optimize storage and computational efficiency and to make the images compatible with the input format of the YOLO model, all images were resized to 680 × 680 pixels. In addition to reducing the computational overhead of the dataset, this scaling guaranteed consistency of the image dimensions, which is essential for preserving data uniformity in the model.

#### 3.1.5. Temporal Sequence Structuring

For flashing traffic lights, the frames were organized into sequences, each containing a fixed number of images representing a specific traffic light state. For example, a flashing red sequence alternated between consecutive “off” and “on” frames, with all frames labeled as flash red. In contrast, a solid red sequence consisted entirely of consecutive “on” frames, all labeled as red. This sequence-based organization enables the LSTM model to effectively capture temporal dependencies within each sequence, which is particularly important for recognizing flashing states. The sequences were then divided into three subsets: 70% for training, 15% for validation, and 15% for testing. These steps ensured that the dataset met the requirements of the proposed traffic light detection and classification model, providing a robust foundation for both model training and systematic evaluation.

To further illustrate the scope of the dataset, our dataset was constructed from 55 real-world videos (15–60 min each, recorded at 50 fps) of intersections in Starkville, MS, and the Mississippi State University campus, as well as 12 simulated videos (1–2 h each) generated in RoadRunner with flashing cycles of 2, 3, and 4 s. For temporal model training, the videos were segmented into frames, and the frames were organized into 2000 sequences (400 per class across the five classes: red, green, yellow, flash red, and flash yellow). Each sequence contained 27 frames, resulting in a total of 54,000 frames. The choice of 27 frames provided sufficient temporal information to capture the “on” and “off” flashing pattern while maintaining computational efficiency. Although the raw video footage is much larger, the curated dataset was intentionally designed to be temporally structured, balanced, and computationally manageable for sequence-based model training. Table 2 gives the detailed specifications of the custom dataset.

### 3.2. Feature Extraction with ResNet-18

The feature extraction process was designed to focus exclusively on the Region of Interest (ROI) within each frame, ensuring the model concentrated on the localized traffic light areas while eliminating irrelevant background information. Using YOLOv10 annotations, precise bounding box coordinates for traffic lights were identified, and the corresponding ROIs were cropped from each frame. These ROIs were resized to 224 × 224 pixels, normalized using ImageNet mean and standard deviation, and transformed into a tensor format compatible with ResNet-18. A pre-trained ResNet-18 model, with its fully connected layer removed, was employed to extract high-dimensional spatial features. This modification produced compact meaningful representations of traffic lights, reducing computational complexity while retaining critical visual information essential for classification. To process sequential frames, the extracted features from all frames in each sequence were aggregated into a unified tensor, enabling effective temporal analysis. The corresponding class ID from the sequence’s label file was paired with the extracted features. These feature tensors, alongside their class IDs, were then passed to the LSTM model for classification. By consolidating spatial features from sequential frames, the pipeline effectively captured temporal dependencies, allowing the LSTM to classify traffic light states accurately, including complex flashing patterns.

### 3.3. Temporal Classification with LSTM

A custom LSTM network was used to capture temporal dependencies and categorize traffic light states, including static and flashing patterns, following the extraction of high-dimensional spatial data using ResNet-18. The network architecture, specifically designed for this task, begins with an input layer that processes the sequential features extracted from ResNet-18. It comprises three LSTM layers, each with 128 hidden units, enabling the model to effectively analyze temporal relationships within sequences. The final hidden state is passed through a fully connected output layer, which maps the hidden states to class probabilities, providing predictions for five traffic light states: red, green, yellow, flash red, and flash yellow. This architecture integrates spatial and temporal features to deliver robust and accurate traffic light classification.

### 3.4. Model Training and Validation

To guarantee optimal performance, a systematic procedure was used to train and evaluate the LSTM model. Three subsets of the dataset were created: training (70%), validation (15%), and testing (15%). With a learning rate of 0.001, the Adaptive Moment Estimation (Adam) optimizer was used to train the model for up to 83 epochs. To avoid overfitting and unnecessary training, early stopping with a 5-epoch patience was used to end training if the validation loss did not improve for five consecutive epochs. The objective function used for this process was cross-entropy loss.

### 3.5. Testing and Evaluation

In order to replicate real-world conditions, the trained LSTM model was evaluated using the test dataset, which was not used for training or validation. In order to predict traffic light states, the model was fed the extracted features from the test sequences. The predictions and the actual labels were then compared. To evaluate how well the model classified both static and flashing traffic lights, the overall F1-score, precision, and recall were calculated. This assessment demonstrated the resilience of the model and highlighted areas where it can be strengthened to handle a range of situations. Furthermore, the suggested model was evaluated on both real and simulated traffic videos with varying flashing rates, demonstrating outstanding performance that confirmed its dependability and practicality in real-world scenarios.

To evaluate the performance of the proposed framework, several evaluation metrics were used, including precision, recall, F1-score, and mAP. Precision is a metric that evaluates the ability of machine learning models to correctly predict the positive class. It can be calculated by dividing the true positive samples by the sum of true positive and false positive samples, as shown in Equation (1) [71,72].

(1) $$\mathrm{Precision}=\frac{\mathrm{TP}}{\mathrm{TP}+\mathrm{FP}}$$

rIn Equation (1), TP means true positive, and FP means false positive. True positive indicates the number of positive instances that the model correctly predicts as positive. False positive is the number of positive instances that the model incorrectly predicts as positive.

Recall reflects a model’s effectiveness in identifying all the actual positive cases within the dataset. It is calculated by dividing the number of true positive predictions by the sum of both true positive and false negative values, as represented in Equation (2) [71,72].

(2) $$\mathrm{Recall}=\frac{\mathrm{TP}}{\mathrm{TP}+\mathrm{FN}}$$

In Equation (2), FN means false negative, and it is defined as the number of positive instances that are incorrectly predicted as negative.

The F1-score is an evaluation metric that combines precision and recall, as shown in Equation (3), where it is widely used to assess the performance of a machine learning model, especially when the dataset is imbalanced, meaning one class appears more frequently than others. The F1-score ranges from 0 to 1, where 1 represents the best possible value, and 0 represents the worst [72].

(3) $$F1=\frac{2PR}{P+R}$$

Here, in Equation (3), P is denoted as precision, and R is denoted as recall. The mAP is the mean of average precision, which is the area under the precision–recall curve, and it is calculated using Equation (4) [71,73].

(4) $$\mathrm{mAP}=\int_{0}^{1}P\left(R\right)\;dR$$

## 4. Experimental Setup

The experimental environment and specifications used in this work to preprocess the dataset and to conduct the experiment are shown in Table 3.

## 5. Results and Discussion

### 5.1. Traffic Light Detection Model

The YOLOv10 model, a single-stage object detection architecture, was used to detect traffic lights in recorded video sequences on a frame-by-frame basis as part of the experimental evaluation. A compact and efficient variant of the YOLOv10 architecture, YOLOv10n, was developed for fast inference and low computational resource requirements, making it suitable for real-time applications [25]. Even without an explicit tracking method (such as SORT or DeepSORT), the frame-by-frame detection demonstrated good temporal consistency, with bounding boxes appearing stable and accurately aligned with the traffic light positions throughout the video. The model was trained and evaluated using a custom dataset containing traffic light states—red, green, yellow, flash red, and flash yellow—captured from various intersections in Starkville and on the Mississippi State University campus, along with a simulation-based traffic light dataset.

YOLOv10 is available in six versions: YOLOv10n (nano), YOLOv10s (small), YOLOv10m (medium), YOLOv10b (balanced), YOLOv10l (large), and YOLOv10x (extra-large) [25]. Each version is designed for different application requirements, with YOLOv10n being the fastest but lower in accuracy, while YOLOv10x is the most accurate but requires the longest training time. In this study, we tested three versions of YOLO—nano, medium, and large—as shown in Table 4.

According to Table 4, the large version achieves slightly better detection performance than the medium and nano versions, with a mAP of 99.9%. The medium and nano versions follow with mAP values of 99.4% and 99.2%, respectively. However, because the large version is computationally more demanding, and the difference in mAP among the large, medium, and nano versions is minimal, we chose the nano version to meet our objective of developing a lightweight model suitable for real-time operation.

### 5.2. Feature Extractor Model

The performance of machine learning models may be hampered by the redundant or unnecessary information often found in raw data [74]. By identifying the most informative features, feature extraction helps to reduce the complexity of the data and enhances the efficiency and accuracy of the downstream classification task [74]. Three convolutional neural network (CNN) architectures—ResNet-18, MobileNetV3 [75], and EfficientNet-B0 [76]—were assessed in this experiment as feature extractors. In order to extract spatial information from the detected traffic light regions, these models were incorporated into the traffic light classification pipeline. The performance of each model was assessed using precision, recall, and F1-score, as shown in Table 5.

The higher the precision, recall, and F1-score values, the better the performance of the model. With a precision of 0.94, recall of 0.97, and an F1-score of 0.95, ResNet-18 outperformed the other models in the test, demonstrating its potent capacity to extract significant features for accurate classification. EfficientNet-B0 and MobileNetV3 also produced competitive results, although their F1 and recall scores were slightly lower. These findings demonstrate that ResNet-18 is the best feature extractor for this use case. It provides a reasonable trade-off between computing efficiency and accuracy, which makes it a good option for real-time traffic light classification systems.

### 5.3. Performance Comparison: LSTM, GRU, and Vanilla RNN with YOLOv10n-ResNet-18

In this experiment, we looked at how well three different types of RNN cells performed when combined with the YOLOv10n-ResNet18 pipeline for the temporal classification of traffic light states across frame sequences: LSTM, Gated Recurrent Unit (GRU) [77,78], and vanilla RNN [79]. Each model received features extracted by ResNet-18 from detected traffic light regions and learned temporal patterns across sequences. The results, presented in Table 6, demonstrate that the LSTM model outperformed the others in terms of precision (0.94), recall (0.97), and F1-score (0.95). These findings highlight how well LSTM captures long-term temporal dependencies, which makes it ideal for sequential tasks like classifying flashing traffic lights.

The precision (0.91), recall (0.94), and F1-score (0.92) were all marginally lower for the GRU model. With fewer parameters and less training time than LSTM, GRU accomplished a favorable trade-off between performance and model complexity. On the other hand, the vanilla RNN model performed the worst out of the three, with precision (0.88), recall (0.92), and F1-score (0.90). These findings align with the well-known shortcomings of basic RNNs in managing long-term dependencies due to vanishing gradient problems.

### 5.4. Tuning the Number of LSTM Layers

The model depth is determined by the number of LSTM layers. Increasing the number of layers generally enhances the test accuracy up to a certain threshold, referred to as the saturation point. In our case, this point was reached with three hidden layers. This behavior aligns with observations in deep neural networks. However, adding more layers also increases the inference time. As shown in Table 7, the model with three LSTM layers provides the best trade-off between accuracy and computational efficiency. Therefore, this configuration was selected for our proposed system.

### 5.5. Optimizer Tuning

The objective of using optimizers in neural networks is to adjust model parameters, such as weights and biases, during training to minimize the loss function. In this experiment, we evaluated the performance of several optimization algorithms—Adam, SGD, and Root Mean Squared Propagation (RMSProp)—to determine which is most effective for the proposed model. The Adam optimizer outperforms the other two optimizers, achieving precision, recall, and F1-score values of 0.96, 0.97, and 0.96, respectively, on the testing dataset.

### 5.6. Learning Rate Tuning

Learning rate tuning is a key hyperparameter that controls how much the model’s weights are adjusted during training in response to the estimated error. It determines the step size at each iteration toward minimizing the loss function. If the learning rate is too high, the model may overshoot the optimal solution and fail to converge. Conversely, if the learning rate is too low, the model may get stuck in a local minimum and converge very slowly. Based on the results presented in Table 8, we experimented with two learning rates: 0.001 and 0.0001. The learning rate of 0.001 yielded the best performance; therefore, we selected it as the default learning rate in our proposed system.

**Figure 4:**
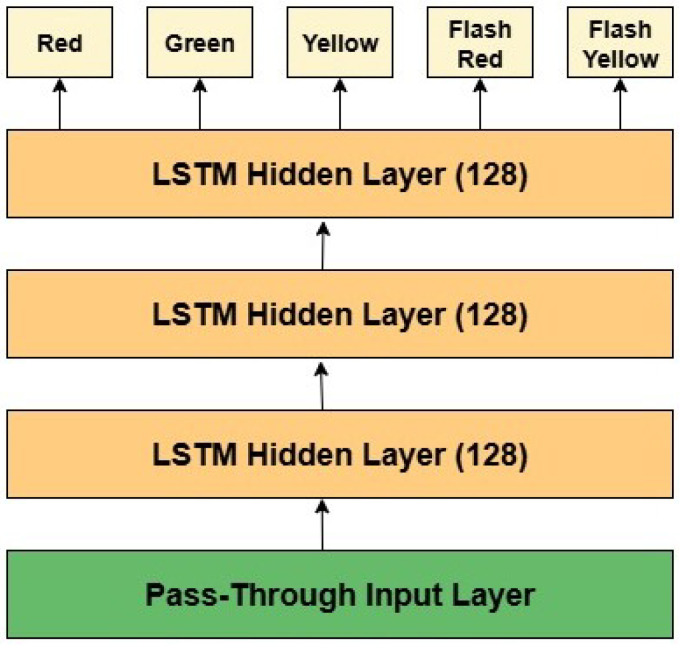
The architecture of the proposed LSTM model.

After conducting extensive trials, testing, and experimentation with various hierarchies and parameter configurations of the base pipeline, we adopted the best-performing configuration from previous experiments as the proposed system for traffic light detection and recognition. The architecture of the proposed model comprises three main components: YOLOv10n for traffic light detection, ResNet-18 for feature extraction, and an LSTM network for classifying traffic light states into red, green, yellow, flash red, and flash yellow. The LSTM model consists of three layers, each containing 128 neurons, as illustrated in Figure 4. Table 9 presents the parameter values of the proposed model that achieved the best results.

### 5.7. Traffic Light State Classification Results

The classification performance of FlashLightNet across individual traffic light states is summarized in Table 10. In recognizing red and yellow lights, the model received perfect scores; its precision, recall, and F1-score all reached 1.00, demonstrating perfect identification of these two classes. With a precision of 0.97, recall of 0.99, and F1-score of 0.98, the green light class likewise did well, demonstrating a high degree of accuracy with only a few misclassifications. The model continued to function reliably even in the more difficult flashing light states. While the flash yellow class scored somewhat lower with precision of 0.92, recall of 0.92, and F1-score of 0.92, the flash red class obtained precision of 0.92, recall of 0.93, and an F1-score of 0.92. The slightly lower F1-scores for the flash red and flash yellow light categories (0.92 each), compared to the static states, can be attributed to the challenges inherent in their dynamic nature. Firstly, the periodic alternation between illuminated (“on”) and non-illuminated (“off”) frames within a single flashing class introduces significant intra-class visual variance, making consistent feature learning more challenging than for static lights. Secondly, the model’s reliance on temporal context poses a challenge; when state transitions occur near the boundaries of an input sequence, the resulting incomplete temporal context can lead to ambiguous pattern recognition and reduced classification accuracy. Finally, real-world environmental factors such as glare, motion blur, and variable lighting can distort the appearance of “off” frames, increasing the risk of misclassifying them as a static red or yellow signal rather than part of a flashing sequence. With a precision of 0.96, recall of 0.97, and F1-score of 0.96, the model’s overall average performance across all five classes was strong, indicating its robustness and reliability in real-world traffic light state recognition scenarios.

**Figure 5:**
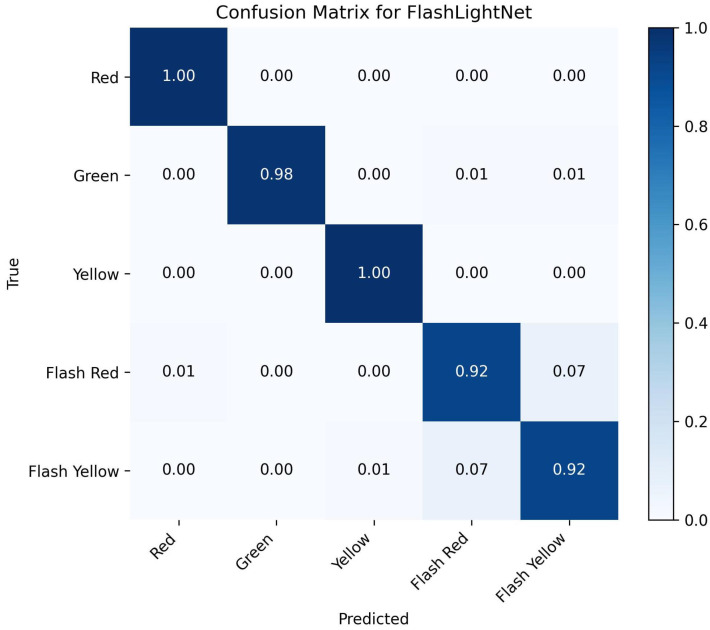
Confusion matrix showing the classification performance of the FlashLightNet Model.

The confusion matrix in Figure 5 illustrates the classification performance of FlashLightNet across five traffic signal states. The results show that the model achieves almost perfect recognition of static signals: all red and yellow samples were classified correctly, while green signals reached an accuracy of 98%, with only a small fraction misclassified as flashing states. For the more challenging flashing categories, FlashLightNet correctly identified 92% of flash red cases, though about 7% were mistaken for flash yellow. Likewise, 92% of flash yellow signals were recognized accurately, with 7% confused with flash red. These findings indicate that while static signals are detected with near-complete reliability, the alternating nature of flashing lights introduces additional complexity. Even so, maintaining over 92% accuracy for both flashing categories shows that the framework is not only robust but also capable of effectively capturing temporal dependencies that are often overlooked by frame-by-frame approaches.

Table 11 presents a comparison between the proposed system (FlashLightNet) and state-of-the-art deep learning models that support real-time performance. Since there are no existing studies that address the recognition of flashing traffic lights, the comparison is limited to static traffic light recognition involving the red, green, and yellow classes. As shown in Table 11, the proposed system outperforms the existing methods in terms of precision, recall, and F1-score.

Based on the above table, the proposed system achieved the best performance in the real-time detection and recognition of static traffic lights, attaining precision, recall, and F1-score values of 0.99, 1.00, and 0.99, respectively. These results demonstrate its superior capability for accurate and robust detection and classification. The second-best performance was obtained by the method developed by Chen and Lin [64], which utilized YOLOv7 combined with ensemble learning and color-based data augmentation. Their approach achieved precision, recall, and F1-score values of 0.96, 0.99, and 0.97, indicating highly reliable detection performance. The method proposed by Rahman et al. [10], which employed YOLOv10n, achieved precision, recall, and F1-score values of 0.98, 0.95, and 0.96, respectively. In contrast, the method by Niu et al. [63], which used YOLOv5s for detection in combination with AlexNet for classification, demonstrated comparatively weaker performance, achieving precision, recall, and F1-score values of 0.91, 0.96, and 0.93, respectively. These results highlight the effectiveness of integrating YOLOv10n for detection, ResNet-18 for feature extraction, and LSTM for temporal classification to significantly enhance the overall model performance.

**Figure 6:**
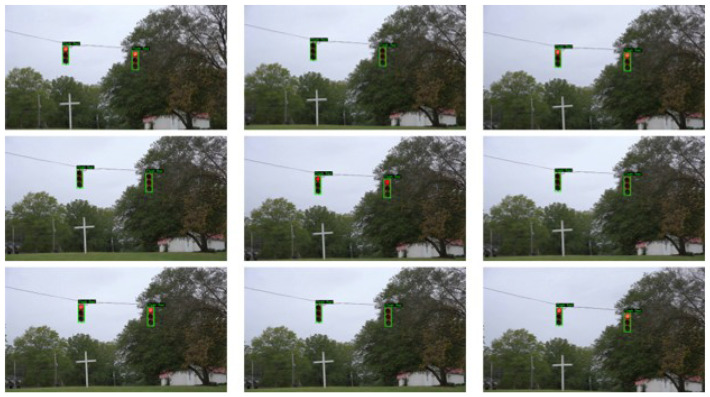
Sample detection and recognition of a flashing traffic light state using the proposed FlashLightNet framework. The input video was captured at 50 fps using a Sony a6500 camera. For model processing, each frame was resized to 680 × 680 pixels. The model consistently labels both “on” and “off” frames as “Flash Red” demonstrating the ability of the LSTM module to preserve temporal context and prevent misclassification of OFF frames. Green bounding boxes indicate detected traffic lights, with the predicted class label displayed above the bounding box.

**Figure 7:**
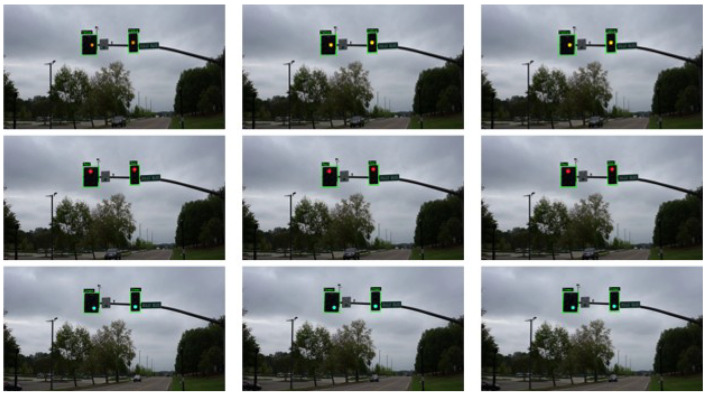
Sample detection and recognition of static traffic light states (red, green, and yellow) using the proposed FlashLightNet framework. Input frames were resized to 680 × 680 pixels, while the input video was recorded at 50 fps with an original resolution of 1920 × 1080. The model demonstrates high performance in recognizing static states, drawing bounding boxes around the detected lights with the predicted class label shown above each detection. These results illustrate the framework’s robustness across both static and flashing signals, as shown in Figure 6.

According to Figure 6 and Figure 7, which show two test samples of detecting and classifying flashing and static traffic lights using the proposed framework, the suggested FlashLightNet performs exceptionally well in both scenarios. Figure 6 displays a sequence of consecutive flash red frames, a particularly challenging situation due to the intermittent nature of the signal. The model consistently identifies each frame as “Flash Red” regardless of whether the light is currently illuminated or off, demonstrating its ability to capture temporal patterns and maintain semantic consistency over time. The incorporation of an LSTM module, which analyzes the sequential frames and successfully models the temporal connections between successive images, is responsible for this reliable detection. Consequently, the model manages to avoid misclassifying “off” frames, a common problem in conventional frame-by-frame classification systems.

In addition, Figure 7 presents a group of consecutive frames representing static traffic light states, including red, green, and yellow. The model achieves outstanding performance in detecting traffic lights by drawing green bounding boxes around the ROIs and correctly classifying each state by labeling the traffic light state above the corresponding bounding box. The proposed model’s ability to accurately distinguish between static and flashing traffic signal states supports its potential use in real-time traffic decision-making systems, such as AVs or intelligent traffic monitoring infrastructure.

### 5.8. Robustness Against Occlusion

In addition to classifying static and flashing signals, a critical requirement for real-world deployment is robustness to transient occlusions, such as those caused by standing behind a large truck or by environmental obstacles. While the current frame-level detector (YOLOv10) may fail to locate a traffic light in a single occluded frame, our proposed system (FlashLightNet) provides inherent resilience against such short-term failures. The LSTM network effectively leverages the historical context from the immediate sequence of frames, maintaining state classification based on the pre-occlusion visual evidence and promptly confirming the state once the occlusion clears. This temporal smoothing prevents misclassifications that could result from frame-by-frame analysis. However, it is important to note that this approach is primarily effective for partial or short-duration occlusions. Performance would understandably degrade under sustained and complete occlusion, where no visual data are available for an extended period. Addressing this limitation is a key focus of our future work, which will explore the integration of contextual data from pre-mapped intersection layouts and V2I communication to predict light states during long periods of invisibility.

### 5.9. Real-Time Requirements Evaluation

In this study, we define real time as the ability of the system to process video input and produce a decision within strict temporal constraints, typically measured in terms of frame rate (≥50 fps), inference latency (≤20 ms per frame), and total decision latency (≤600 ms). These thresholds align with the prior literature on real-time traffic perception systems and ensure that flashing light states can be reliably detected in a practical deployment scenario. For example, ViTLR [80], a video-based end-to-end neural network, achieved real-time traffic light recognition at just >25 fps, whereas Jayasinghe et al. [81] achieves an inference speed of 63 fps in recognizing traffic signs and static traffic lights, but without explicitly addressing flashing signals. Similarly, a lightweight recognition system [82] demonstrated real-time operation at 30 fps. In contrast, our stricter thresholds impose tighter latency constraints, which provide a practical and conservative definition of real-time performance. The real-time performance metrics of the proposed traffic light recognition system are summarized in Table 12.

Our system achieves a camera capture rate of 50 fps (20 ms per frame), with a flashing detection window of 27 frames, resulting in an initial detection delay of 540 ms. Including inference time (15.60 ms per frame), the total decision latency is 555.60 ms, which remains within the 600 ms real-time requirement. Once the initial flashing state is detected, the system maintains a throughput of 67.15 fps, surpassing the minimum 50 fps threshold. The Continuous Detection Latency (FIFO) is also only 14.18 ms per decision, which indicates the system’s ability to keep up with fast responses despite continuously running. These results demonstrate that the proposed framework satisfies real-time constraints and can robustly handle both static and flashing traffic light states in continuous operation.

These results demonstrate the model’s ability to classify and adapt across a wide range of conditions. It delivers strong performance in both static scenarios—such as fixed traffic light states—and temporal sequences involving rapid state transitions, such as flashing signals. Moreover, the model’s detections remain accurate and stable despite variations in camera angles, lighting conditions, and background context, highlighting its effective generalization capabilities. This high performance underscores the effectiveness of combining YOLOv10n’s fast and precise spatial detection, ResNet-18’s deep feature extraction, and LSTM’s sequence modeling to operate successfully in both temporally dynamic and visually stable environments. The model also demonstrates a high capacity for understanding temporal context in real time, which is a critical requirement for intelligent transportation systems. Overall, the above results support the model’s reliability, accuracy, and applicability in diverse real-world settings with intricate traffic patterns. This makes the proposed framework a promising solution that can be effectively applied in autonomous navigation systems and advanced driver assistance systems (ADAS).

## 6. Conclusions

This study presents FlashLightNet, an end-to-end deep learning framework for real-time traffic light detection and classification, uniquely addressing both static (red, green, and yellow) and flashing (flash red and flash yellow) signal states. The proposed architecture integrates three complementary modules: YOLOv10n for accurate and lightweight object detection, ResNet-18 for deep spatial feature extraction, and LSTM network to capture and classify temporal patterns critical to recognizing flash states.

Unlike many prior approaches that overlook the complexity and intermittency of flashing signals, our framework was specifically designed to overcome this limitation. The system was trained and evaluated on a custom dataset captured from various intersections throughout Starkville, Mississippi, and the Mississippi State University campus, as well as a simulated video dataset for traffic light states at several intersections generated using RoadRunner. This dataset incorporates diverse lighting conditions, environmental noise, and traffic behaviors to reflect real-world complexities. The proposed framework achieved an outstanding mAP of 99.2% for detection and an F1-score of 96% for classification, clearly demonstrating its robustness, generalization capability, and real-time performance. In particular, it has achieved high classification accuracy in all five classes of traffic lights, including the more challenging flashing states. By employing sequence-based input structures and a tailored LSTM model, the system successfully mitigates issues such as misclassification during OFF frames, a known limitation in traditional frame-based models.

Furthermore, a comparative evaluation with alternative temporal models—namely, GRU and vanilla RNN—reinforced the superiority of the LSTM-based classification architecture in capturing long-range dependencies and temporal consistency. Among all tested feature extractors, ResNet-18 provided the best balance between accuracy and computational efficiency, further enhancing the system’s suitability for deployment in resource-constrained environments such as edge devices, intelligent intersections, or autonomous driving platforms.

We recognize that the optimal solution for AV involves direct digital communication (V2I) with traffic infrastructure. However, widespread deployment of such infrastructure is a long-term goal. Our work provides a critical capability for the current and extended transition period, where vehicles must navigate a world dominated by outdated traffic light systems. The ability to accurately interpret flashing lights is critically important, as these states often indicate dangerous or unusual conditions, such as intersections with malfunctioning lights or preempting emergency vehicles. We argue that perception systems like FlashLightNet will remain important not only as a primary solution for current infrastructure, but also as a vital backup system in the future V2I, ensuring safety and security from potential communications failures.

Although the system has demonstrated remarkable performance, several avenues remain for future research. Future work includes incorporating contextual data such as vehicle trajectory, speed, GPS location, and surrounding traffic flow to further enhance decision-making in dynamic urban environments, extending the model to handle additional signal types (e.g., arrow, pedestrians, and countdown lights). In the future, integrating sensor fusion, such as pre-mapped traffic light locations via GPS combined with camera input, LiDAR, radar, or V2X communication, will help mitigate limitations in low-visibility or occluded scenarios.

In conclusion, the proposed YOLOv10n–ResNet18–LSTM framework significantly advances the field of intelligent traffic light recognition by providing a robust, scalable, and real-time solution capable of interpreting complex signal states. This work not only fills a critical research gap in flashing light classification but also lays a solid foundation for the next generation of autonomous driving and smart transportation systems.

## Figures and Tables

**Table 1:** Summary of traffic light detection and recognition studies: A comparative analysis of datasets, methods, and model performance.

Work Type	Detect and Classify Static Traffic Light	Detect and Classify Flashing Traffic Light	Real Time	Dataset	Model Used
Rao et al. [9]	✓	✗	✓	LISA Cropped Traffic Light dataset	ResNet50 (Residual Networks)
Rahman et al. [10]	✓	✗	✓	S2TLD (Small-scale Traffic Light dataset)	YOLOv8s
De Guia et al. [6]	✓	✗	✓	Custom TLR (700 K images) and TSD (100 K images) datasets	YOLOv7 (primary), YOLOv5, YOLOv3
Kulkarni et al. [65]	✓	✗	✗	Custom dataset (1237 images, Indian traffic lights)	Faster R-CNN with Inception-V2
Niu et al. [63]	✓	✗	✓	Custom dataset (4200 images, including low-light/foggy conditions)	YOLOv5s for detection and AlexNet for classification
Ajagbe et al. [66]	✓	✗	✗	LaRA Traffic Lights Recognition dataset	Faster R-CNN, Fast R-CNN, R-CNN, R-FCN, SPPNet
Chen and Lin [64]	✓	✗	✓	Private + relabeled public datasets (Lara, WPI, TL-Seoul), 34,388 frames	YOLOv7 + Ensemble Learning + Color-Based Data Augmentation
Our Work (FlashLightNet)	✓	✓	✓	Custom dataset (Starkville, Mississippi State University, and simulated dataset)	YOLOv10n-ResNet18-LSTM

**Note:** The symbol ✓ indicates the feature is present or implemented, while ✗ indicates it is absent or not implemented.

**Table 2:** Detailed specifications of the custom dataset.

Component Category	Specification	Details
Data Source	Real-world Videos	55 videos, 15–60 min each, 50 fps
	Simulated Videos (RoadRunner)	12 videos, 1–2 h each, variable flashing rates (2, 3, 4 s)
Processed Dataset	Total Sequences	2000
	Sequences per Class	400 (for each of the 5 classes)
	Frames per Sequence	27
	Total Frames	54,000 (2000 × 27)
	Classes	Red, Green, Yellow, Flash Red, and Flash Yellow
Data Split	Training	70% (1400 sequences)
	Validation	15% (300 sequences)
	Testing	15% (300 sequences)

**Table 3:** Experimental environment specifications.

Components	Specification
GPU	4× NVIDIA A100 80GB PCIe
CPU	Intel^®^ Xeon^®^ Platinum 8362 CPU @ 2.80 GHz, 32 cores, 64 threads (2 threads/core, 2 sockets)
RAM	64 GB DDR4
Operating System	Linux CentOS Stream 9—Kernel: 5.14.0-284.11.1.el9_2.x86_64
Deep Learning Frameworks	Python 3.9.21, torch 2.5.1+cu121, torchvision 0.20.1+cu121, torchaudio 2.5.1+cu121, ultralytics 8.1.34, opencv-python 4.10.0.84, numpy 1.26.4, pandas 2.2.3, scikit-learn 1.5.2, scikit-image 0.24.0, matplotlib 3.9.2, seaborn 0.13.2, albumentations 1.4.0, tqdm 4.67.1, triton 3.1.0.

**Table 4:** Performance of several versions of YOLOv10 in terms of mAP.

Yolo10 Version	mAP	Training Time (Hours)
Nano	99.2	7.45
Medium	99.4	10.99
Large	99.9	24.61

**Table 5:** Performance comparison of feature extractors in terms of precision, recall, and F1-score.

Feature Extractor	Precision	Recall	F1-Score
ResNet-18	0.94	0.97	0.95
MobileNetV3	0.93	0.90	0.91
EfficientNet-B0	0.86	0.89	0.87

**Table 6:** Performance comparison of LSTM, GRU, and vanilla RNN with YOLOv10n-ResNet18 in terms of precision, recall, and F1-score.

RNN Type	Precision	Recall	F1-Score
GRU	0.91	0.94	0.92
Vanilla RNN	0.88	0.92	0.90
LSTM	0.94	0.97	0.95

**Table 7:** Performance comparison of models with different numbers of layers in terms of precision, recall, and F1-score.

Number of Layers	Precision	Recall	F1-Score
2	0.94	0.97	0.95
3	0.96	0.97	0.96
4	0.84	0.94	0.89

**Table 8:** Performance comparison between the two learning rates in terms of precision, recall, and F1-score.

Learning Rate	Precision	Recall	F1-Score
0.001	0.96	0.97	0.96
0.0001	0.88	0.85	0.86

**Table 9:** The values of the proposed model parameter that achieved the best results.

Parameters	Value
YOLO	Version 10 (Nano)
YOLO Parameters	2.3 million
ResNet	18 Layers
Number of LSTM Layers	3
LSTM Parameters	593,541
Number of Nodes in Each Layer	128
Optimizer	Adam
Learning Rate	0.001
Evaluation Metrics	Precision, Recall, F1-score, and mAP

**Table 10:** Classification performance for each traffic light state in terms of precision, recall, and F1-score.

Class	Precision	Recall	F1-Score
Red	1.00	1.00	1.00
Green	0.97	0.99	0.98
Yellow	1.00	1.00	1.00
Flash Red	0.92	0.93	0.92
Flash Yellow	0.92	0.92	0.92
Average	0.96	0.97	0.96

**Table 11:** Comparison of the proposed system with state-of-the-art models for static traffic light recognition. Best results are indicated in bold.

Class	Precision	Recall	F1-Score
Niu et al. [63]	0.91	0.96	0.93
De Guia et al. [6]	0.94	0.97	0.95
Rahman et al. [10]	0.98	0.95	0.96
Chen and Lin [64]	0.96	0.99	0.97
**FlashLightNet (Proposed Method)**	**0.99**	**1.00**	**0.99**

**Table 12:** Real-time performance metrics for the proposed traffic light detection system.

Metric	Definition	Measured Value	Real-Time Requirement	Meets Requirement?
Camera Frame Rate (fps)	Number of frames captured per second	50	≥50 fps	Yes
Frame Interval	1/fps	20 ms	-	-
M (Flashing Detection Window)	Number of frames needed to detect flashing	27	-	-
Initial Flashing Detection Delay	M × frame interval	540 ms	≤600 ms	Yes
Inference Time per Frame	Processing time for a single frame	15.60 ms	≤20 ms	Yes
Total Decision Latency	Initial delay + inference time	555.60 ms	≤600 ms	Yes
Continuous Detection Latency (FIFO)	Time per decision during continuous detection after initial flashing recognition	14.18 ms	≤20 ms	Yes
Throughput After First Decision	Fps maintained after initial detection	67.15 fps	≥50 fps	Yes

## Data Availability

The dataset generated and analyzed in this study is not publicly available at this time due to ongoing research activities and pending formal permissions for distribution. However, it can be obtained from the corresponding author upon reasonable request under a formal data sharing agreement. The dataset will be made publicly accessible once the related projects are completed and all necessary approvals have been secured.

## References

[1] Triki N., Karray M., Ksantini M. (2023). A Real-Time Traffic Sign Recognition Method Using a New Attention-Based Deep Convolutional Neural Network for Smart Vehicles. Appl. Sci..

[2] Min W., Liu R., He D., Han Q., Wei Q., Wang Q. (2022). Traffic Sign Recognition Based on Semantic Scene Understanding and Structural Traffic Sign Location. IEEE Trans. Intell. Transp. Syst..

[3] Wang L., Zhou K., Chu A., Wang G., Wang L. (2021). An Improved Light-Weight Traffic Sign Recognition Algorithm Based on YOLOv4-Tiny. IEEE Access.

[4] Jang C., Kim C., Kim D., Lee M., Sunwoo M. Multiple exposure images based traffic light recognition. Proceedings of the 2014 IEEE Intelligent Vehicles Symposium.

[5] Jensen M.B., Philipsen M.P., Møgelmose A., Moeslund T.B., Trivedi M.M. (2016). Vision for looking at traffic lights: Issues, survey, and perspectives. IEEE Trans. Intell. Transp. Syst..

[6] De Guia J.M., Deveraj M. (2024). Development of Traffic Light and Road Sign Detection and Recognition Using Deep Learning. Development.

[7] Lin H.Y., Lin S.Y., Tu K.C. (2024). Traffic Light Detection and Recognition using a Two-Stage Framework from Individual Signal Bulb Identification. IEEE Access.

[8] Yao Z., Liu Q., Fu J., Xie Q., Li B., Ye Q., Li Q. (2024). A coarse-to-fine deep learning based framework for traffic light recognition. IEEE Trans. Intell. Transp. Syst..

[9] Nagendra R., Archana T., Sirisha M., Vishnuvardhan T., Shreya S. (2024). TRAFFIC LIGHT DETECTION AND CLASSIFICATION USING RESNET50. Int. J. HRM Organ. Behav..

[10] Rahman M., Islam F., Ball J.E., Goodin C. (2024). Traffic light recognition and V2I communications of an autonomous vehicle with the traffic light for effective intersection navigation using YOLOv8 and MAVS simulation. Autonomous Systems: Sensors, Processing, and Security for Ground, Air, Sea, and Space Vehicles and Infrastructure 2024.

[11] Fairfield N., Urmson C. Traffic light mapping and detection. Proceedings of the 2011 IEEE International Conference on Robotics and Automation.

[12] Diaz M., Cerri P., Pirlo G., Ferrer M.A., Impedovo D. (2015). A survey on traffic light detection. New Trends in Image Analysis and Processing, Proceedings of the International Conference on Image Analysis and Processing (ICIAP 2015 Workshops), Genoa, Italy, 7–11 September 2015.

[13] Federal Highway Administration (2004). Signalized Intersections: Informational Guide. Chapter One—Introduction. https://international.fhwa.dot.gov/pubs/pl03020/chp01.cfm.

[14] Srinivasan R., Carter D., Eccles K., Persaud B., Lefler N., Lyon C., Amjadi R. (2008). Safety Evaluation of Flashing Beacons at STOP-Controlled Intersections.

[15] Kothuri S., Monsere C., Jashami H., Hurwitz D.S. (2020). Online Survey of Driver Comprehension of the Flashing Yellow Arrow for Right-Turn Signal Indications. J. Transp. Eng. Part A Syst..

[16] Staples B., Chang J. (2020). Vehicle to Infrastructure (V2I) Program: Research, Development, and Deployment Support Conducted Through 2020.

[17] Budan G., Hayatleh K., Morrey D., Ball P., Shadbolt P. (2018). An analysis of vehicle-to-infrastructure communications for non-signalised intersection control under mixed driving behaviour. Analog Integr. Circuits Signal Process..

[18] Mostowfi S., Buttlar W.G. (2020). Vehicle-to-infrastructure and human-to-infrastructure models for smart civil infrastructure systems. AHFE 2020: Advances in Human Aspects of Transportation.

[19] Nokes T., Baxter B., Scammell H., Naberezhnykh D., Provvedi L. (2020). Cost Analysis of V2I Deployment.

[20] Altuijary M. (2020). An Overview of Vehicle-to-Infrastructure Communication Technology. Master’s Thesis.

[21] Hassan H., Sultana T. (2022). Studying the Impacts of Vehicle-to-Infrastructure (V2I) Technologies on Drivers’ Behaviors and Traffic Safety.

[22] Sarlak A., Amin R., Razi A. (2025). Extended Visibility of Autonomous Vehicles via Optimized Cooperative Perception under Imperfect Communication. arXiv.

[23] Gu Y., Kang D. Traffic Signal Recognition and Application Algorithm for the Autonomous Vehicle in V2X Unable Areas. Proceedings of the Eighteenth International Conference on Autonomic and Autonomous Systems (CAS 2022).

[24] Gyawali S., Xu S., Qian Y., Hu R.Q. (2020). Challenges and solutions for cellular based V2X communications. IEEE Commun. Surv. Tutor..

[25] Wang A., Chen H., Liu L., Chen K., Lin Z., Han J., Ding G. (2024). Yolov10: Real-time end-to-end object detection. Adv. Neural Inf. Process. Syst..

[26] He K., Zhang X., Ren S., Sun J. Deep residual learning for image recognition. Proceedings of the IEEE Conference on Computer Vision and Pattern Recognition.

[27] Hochreiter S., Schmidhuber J. (1997). Long Short-Term Memory. Neural Comput..

[28] Omachi M., Omachi S. Traffic light detection with color and edge information. Proceedings of the 2009 2nd IEEE International Conference on Computer Science and Information Technology.

[29] Diaz-Cabrera M., Cerri P., Sanchez-Medina J. Suspended traffic lights detection and distance estimation using color features. Proceedings of the 2012 15th International IEEE Conference on Intelligent Transportation Systems.

[30] Gong J., Jiang Y., Xiong G., Guan C., Tao G., Chen H. The recognition and tracking of traffic lights based on color segmentation and camshift for intelligent vehicles. Proceedings of the 2010 IEEE Intelligent Vehicles Symposium.

[31] Chiang C.C., Ho M.C., Liao H.S., Pratama A., Syu W.C. (2011). Detecting and recognizing traffic lights by genetic approximate ellipse detection and spatial texture layouts. Int. J. Innov. Comput. Inf. Control.

[32] Kim H.-K., Shin Y.-N., Kuk S.-G., Park J.H., Jung H.-Y. (2013). Night-time traffic light detection based on SVM with geometric moment features. Int. Sch. Sci. Res. Innov..

[33] Kim H.K., Park J.H., Jung H.Y. (2011). Effective traffic lights recognition method for real time driving assistance system in the daytime. Int. Sch. Sci. Res. Innov..

[34] Wang C., Jin T., Yang M., Wang B. (2011). Robust and real-time traffic lights recognition in complex urban environments. Int. J. Comput. Intell. Syst..

[35] Diaz-Cabrera M., Cerri P., Medici P. (2015). Robust real-time traffic light detection and distance estimation using a single camera. Expert Syst. Appl..

[36] Diaz-Cabrera M., Cerri P. (2013). Traffic light recognition during the night based on fuzzy logic clustering. Computer Aided Systems Theory—EUROCAST 2013.

[37] De Charette R., Nashashibi F. Real time visual traffic lights recognition based on spot light detection and adaptive traffic lights templates. Proceedings of the 2009 IEEE Intelligent Vehicles Symposium.

[38] Zhang Y., Xue J., Zhang G., Zhang Y., Zheng N. A multi-feature fusion based traffic light recognition algorithm for intelligent vehicles. Proceedings of the 33rd Chinese Control Conference.

[39] Koukoumidis E., Martonosi M., Peh L.S. (2011). Leveraging smartphone cameras for collaborative road advisories. IEEE Trans. Mob. Comput..

[40] Sooksatra S., Kondo T. Red traffic light detection using fast radial symmetry transform. Proceedings of the 2014 11th International Conference on Electrical Engineering/Electronics, Computer, Telecommunications and Information Technology (ECTI-CON).

[41] Nienhüser D., Drescher M., Zöllner J.M. Visual state estimation of traffic lights using hidden Markov models. Proceedings of the 13th International IEEE Conference on Intelligent Transportation Systems.

[42] Lindner F., Kressel U., Kaelberer S. Robust recognition of traffic signals. Proceedings of the IEEE Intelligent Vehicles Symposium.

[43] Barnes D., Maddern W., Posner I. Exploiting 3D semantic scene priors for online traffic light interpretation. Proceedings of the 2015 IEEE Intelligent Vehicles Symposium (IV).

[44] Cai Z., Li Y., Gu M. Real-time recognition system of traffic light in urban environment. Proceedings of the 2012 IEEE Symposium on Computational Intelligence for Security and Defence Applications.

[45] Haltakov V., Mayr J., Unger C., Ilic S. (2015). Semantic segmentation based traffic light detection at day and at night. Proceedings of the German Conference on Pattern Recognition, Aachen, Germany, 7–10 October 2015.

[46] Redmon J., Divvala S., Girshick R., Farhadi A. You only look once: Unified, real-time object detection. Proceedings of the IEEE Conference on Computer Vision and Pattern Recognition.

[47] Girshick R. Fast R-CNN. Proceedings of the IEEE International Conference on Computer Vision.

[48] Liu W., Anguelov D., Erhan D., Szegedy C., Reed S., Fu C.Y., Berg A.C. (2016). SSD: Single shot multibox detector. Computer Vision—ECCV 2016, Proceedings of the 14th European Conference, Amsterdam, The Netherlands, 11–14 October 2016.

[49] Ennahhal Z., Berrada I., Fardousse K. Real time traffic light detection and classification using deep learning. Proceedings of the 2019 International Conference on Wireless Networks and Mobile Communications (WINCOM).

[50] Wang Q., Zhang Q., Liang X., Wang Y., Zhou C., Mikulovich V.I. (2021). Traffic lights detection and recognition method based on the improved YOLOv4 algorithm. Sensors.

[51] Possatti L.C., Guidolini R., Cardoso V.B., Berriel R.F., Paixão T.M., Badue C., De Souza A.F., Oliveira-Santos T. Traffic light recognition using deep learning and prior maps for autonomous cars. Proceedings of the 2019 International Joint Conference on Neural Networks (IJCNN).

[52] Abduljabbar R.L., Dia H., Tsai P.W., Liyanage S. (2021). Short-term traffic forecasting: An LSTM network for spatial-temporal speed prediction. Future Transp..

[53] Gopali S., Abri F., Siami-Namini S., Namin A.S. (2021). A comparative study of detecting anomalies in time series data using LSTM and TCN models. arXiv.

[54] Yu Y., Zeng X., Xue X., Ma J. (2022). LSTM-based intrusion detection system for VANETs: A time series classification approach to false message detection. IEEE Trans. Intell. Transp. Syst..

[55] Khodairy M.A., Abosamra G. (2021). Driving behavior classification based on oversampled signals of smartphone embedded sensors using an optimized stacked-LSTM neural networks. IEEE Access.

[56] Nan M., Trăscău M., Florea A.M., Iacob C.C. (2021). Comparison between recurrent networks and temporal convolutional networks approaches for skeleton-based action recognition. Sensors.

[57] Shiguihara P., Lopes A.D.A., Mauricio D. (2021). Dynamic Bayesian network modeling, learning, and inference: A survey. IEEE Access.

[58] D’Aniello G. (2023). Fuzzy logic for situation awareness: A systematic review. J. Ambient Intell. Humaniz. Comput..

[59] Lu B., Luktarhan N., Ding C., Zhang W. (2021). ICLSTM: Encrypted traffic service identification based on inception-LSTM neural network. Symmetry.

[60] Singh Y.P., Lobiyal D.K. (2023). Automatic prediction of epileptic seizure using hybrid deep ResNet-LSTM model. AI Commun..

[61] Song S., Lam J.C., Han Y., Li V.O. (2020). ResNet-LSTM for Real-Time PM_2.5_ and PM_10_ Estimation Using Sequential Smartphone Images. IEEE Access.

[62] Le-Xuan T., Bui-Tien T., Tran-Ngoc H. (2024). A novel approach model design for signal data using 1DCNN combing with LSTM and ResNet for damaged detection problem. Structures.

[63] Niu C., Li K. (2022). Traffic light detection and recognition method based on YOLOv5s and AlexNet. Appl. Sci..

[64] Chen Y.C., Lin H.Y. Traffic Light Detection and Recognition using Ensemble Learning with Color-Based Data Augmentation. Proceedings of the 2024 IEEE Intelligent Vehicles Symposium (IV).

[65] Kulkarni R., Dhavalikar S., Bangar S. Traffic light detection and recognition for self driving cars using deep learning. Proceedings of the 2018 Fourth International Conference on Computing Communication Control and Automation (ICCUBEA).

[66] Ajagbe S.A., Adegun A.A., Olanrewaju A.B., Oladosu J.B., Adigun M.O. (2023). Performance investigation of two-stage detection techniques using traffic light detection dataset. IAES Int. J. Artif. Intell. (IJ-AI).

[67] Al-Haija Q.A., Smadi M.A., Zein-Sabatto S. Multi-class weather classification using ResNet-18 CNN for autonomous IoT and CPS applications. Proceedings of the 2020 International Conference on Computational Science and Computational Intelligence (CSCI).

[68] Abhishek A.V.S., Gurrala V.R. (2022). Improving model performance and removing the class imbalance problem using augmentation. Int. J. Adv. Res. Eng. Technol. (IJARET).

[69] Ramzan F., Khan M.U.G., Rehmat A., Iqbal S., Saba T., Rehman A., Mehmood Z. (2020). A deep learning approach for automated diagnosis and multi-class classification of Alzheimer’s disease stages using resting-state fMRI and residual neural networks. J. Med Syst..

[70] Azzouni A., Pujolle G. (2017). A long short-term memory recurrent neural network framework for network traffic matrix prediction. arXiv.

[71] Davis J., Goadrich M. The relationship between Precision-Recall and ROC curves. Proceedings of the 23rd International Conference on Machine Learning.

[72] Weber M., Wolf P., Zöllner J.M. DeepTLR: A single deep convolutional network for detection and classification of traffic lights. Proceedings of the 2016 IEEE Intelligent Vehicles Symposium (IV).

[73] Zhu M. Recall, Precision and Average Precision. https://datascience-intro.github.io/1MS041-2022/Files/AveragePrecision.pdf.

[74] Guyon I., Elisseeff A. (2003). An introduction to variable and feature selection. J. Mach. Learn. Res..

[75] Howard A., Sandler M., Chu G., Chen L.C., Chen B., Tan M., Wang W., Zhu Y., Pang R., Vasudevan V. Searching for MobileNetV3. Proceedings of the IEEE/CVF International Conference on Computer Vision.

[76] Tan M., Le Q. Efficientnet: Rethinking model scaling for convolutional neural networks. Proceedings of the International Conference on Machine Learning (PMLR 97).

[77] Cho K., Van Merriënboer B., Gulcehre C., Bahdanau D., Bougares F., Schwenk H., Bengio Y. (2014). Learning phrase representations using RNN encoder-decoder for statistical machine translation. arXiv.

[78] Cho K., Van Merriënboer B., Bahdanau D., Bengio Y. (2014). On the properties of neural machine translation: Encoder-decoder approaches. arXiv.

[79] Yi D., Kim I., Bu S. (2024). Variations of Training Process in Vanilla Recurrent Neural Network Framework. Neural Netw. World.

[80] Fan M., Kong X., Xu S., Xiong H., Liu X. (2025). Video-based Traffic Light Recognition by Rockchip RV1126 for Autonomous Driving. arXiv.

[81] Jayasinghe O., Hemachandra S., Anhettigama D., Kariyawasam S., Wickremasinghe T., Ekanayake C., Rodrigo R., Jayasekara P. Towards real-time traffic sign and traffic light detection on embedded systems. Proceedings of the 2022 IEEE Intelligent Vehicles Symposium (IV).

[82] Choi J., Lee H. (2024). Real-time traffic light recognition with lightweight state recognition and ratio-preserving zero padding. Electronics.

